# *Notes from the Field:* Suspected Outbreak of Trichinellosis Associated with Undercooked Bear Meat — North Carolina, November 2023

**DOI:** 10.15585/mmwr.mm7340a4

**Published:** 2024-10-10

**Authors:** Camden D. Gowler, Nicole Lee, Tammra Morrison, Vivian Mears, Carl Williams, Aaron Fleischauer, Erica Wilson

**Affiliations:** ^1^Epidemic Intelligence Service, CDC; ^2^North Carolina Department of Health and Human Services; ^3^Career Epidemiology Field Officer Program, CDC.

**Summary**

**What is already known about this topic?**

Trichinellosis is a rare parasitic disease; an increasing percentage of recent cases are associated with consumption of wild game meat.

**What is added by this report?**

In November 2023, a presumed outbreak of trichinellosis occurred in western North Carolina, resulting in 10 probable cases. All cases were linked to a gathering where attendees consumed undercooked bear meat.

**What are the implications for public health practice?**

Communication of safe wild game meat preparation is the most effective way to prevent trichinellosis. Diagnostic antibody tests might have poor accuracy, and treatment costs can be substantial. Cooking wild game meat to an internal temperature ≥165°F (≥74°C) is necessary to kill *Trichinella *spp. parasites.

*Trichinella *spp. nematodes are complex life cycle parasites that can cause trichinellosis (also called trichinosis) when humans consume undercooked or raw meat harboring dormant larvae (*1*). Trichinellosis is rare in the United States, largely as a result of changes in pig-raising practices, with most recently reported cases being associated with consumption of wild game meat (*2*). Signs and symptoms include myalgia and fever in 54% of cases and facial swelling in 42% (*2*). Timely identification is important because trichinellosis can be severe; 0.2% of cases are fatal (*1*).

On November 29, 2023, the North Carolina Division of Public Health was alerted to a suspected case of trichinellosis in western North Carolina. The index patient experienced influenza-like signs and symptoms and facial swelling. Further investigation linked this patient to a gathering in early November where undercooked bear meat was served.

## Investigations and Outcomes

Among 34 surveyed attendees at the November 2023 gathering, 22 (65%) reported consuming undercooked bear meat at the gathering; 10 (45%) of these persons experienced clinical signs and symptoms consistent with the 2014 Council of State and Territorial Epidemiologists’ trichinellosis probable case classification* (*3*). Five patients received testing for *Trichinella* immunoglobulin G antibodies; all results were negative. However, confirmatory diagnosis requires additional testing of convalescent samples, and none of those receiving testing returned for convalescent serum testing. No bear meat was available for laboratory testing. Data from attendees and medical records from patients were collected and analyzed to guide public health actions. This activity was reviewed by CDC, deemed not research, and was conducted consistent with applicable federal law and CDC policy.^†^

Among the 10 probable cases, nine patients had facial swelling, six had myalgia, and four had documented fever. Median patient age was 17 years (range = 10–40 years). Six probable trichinellosis cases occurred among persons aged ≤18 years. The median incubation period (interval from the implicated meal to documented symptom onset) was 21 days (range = 7–26 days) (Figure).

**FIGURE:**
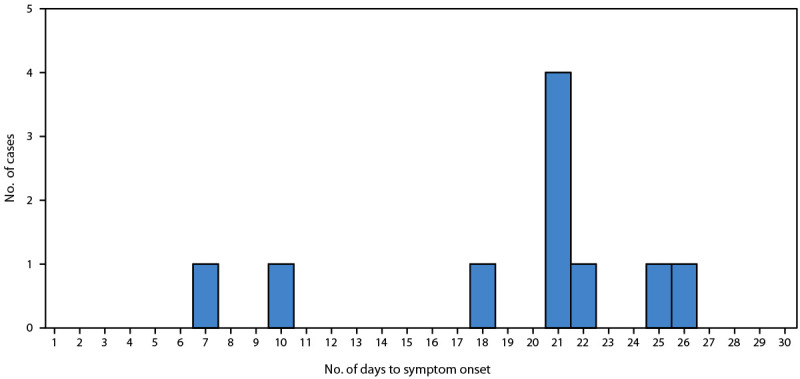
Interval (days) from consumption of undercooked bear meat to symptom onset among persons with probable trichinellosis (N = 10) — North Carolina, November 2023

## Preliminary Conclusions and Actions

North Carolina public health officials identified probable trichinellosis cases based on clinical and epidemiologic criteria. Although *Trichinella* infections remain rare, thousands of bears are harvested each year in North Carolina (*4*). New *Trichinella* seroprevalence surveys for wild game species might be warranted. A 2022 trichinellosis outbreak associated with undercooked bear meat harvested from Canada resulted in six trichinellosis cases, including cases in two patients who only ate vegetables and were infected by cross-contamination (*5*). Because black bears are common hosts for *Trichinella *spp., communicating methods for properly cooking and preparing wild game meat is important. Cooking game meat to a safe internal temperature (≥165°F [≥74°C]) will kill *Trichinella* spp. and prevent infection, whereas freezing might not be sufficient (*1*).

In severe cases, trichinellosis can result in persistent myalgia or death (*1*). The majority of symptomatic persons in this outbreak were prescribed an antihelminth (albendazole), but use was delayed in some instances. Several patients reported a prohibitively high cost for treatment (approximately $100 per course). Moreover, whether the patient was treated or not, confirming infection through testing of convalescent serum is challenging because acute symptoms have often resolved by the time samples can be collected. Recovered patients might have little incentive to return for testing. Challenges associated with diagnosis and treatment of trichinellosis serve as a reminder for local health departments and wildlife management to communicate safe wild game meat preparation.

## Footnotes

† 45 C.F.R. part 46, 21 C.F.R. part 56; 42 U.S.C. Sect. 241(d); 5 U.S.C. Sect. 552a; 44 U.S.C. Sect. 3501 et seq.
